# Interspecific Plant Interactions Reflected in Soil Bacterial Community Structure and Nitrogen Cycling in Primary Succession

**DOI:** 10.3389/fmicb.2018.00128

**Published:** 2018-02-06

**Authors:** Joseph E. Knelman, Emily B. Graham, Janet S. Prevéy, Michael S. Robeson, Patrick Kelly, Eran Hood, Steve K. Schmidt

**Affiliations:** ^1^Institute of Arctic and Alpine Research, University of Colorado Boulder, Boulder, CO, United States; ^2^Pacific Northwest National Laboratory (U.S. Department of Energy), Richland, WA, United States; ^3^Pacific Northwest Research Station, The United States Forest Service, Olympia, WA, United States; ^4^Department of Biomedical Informatics, University of Arkansas for Medical Sciences, Little Rock, AR, United States; ^5^Department of Natural Sciences, University of Alaska Southeast, Juneau, AK, United States; ^6^Department of Ecology and Evolutionary Biology, University of Colorado Boulder, Boulder, CO, United States

**Keywords:** plant–microbe interactions, primary succession, plant–plant interactions, bacterial community, soil, glacier forefield, nitrogen cycling, plant microbiome

## Abstract

Past research demonstrating the importance plant–microbe interactions as drivers of ecosystem succession has focused on how plants condition soil microbial communities, impacting subsequent plant performance and plant community assembly. These studies, however, largely treat microbial communities as a black box. In this study, we sought to examine how emblematic shifts from early successional *Alnus viridus* ssp. *sinuata* (Sitka alder) to late successional *Picea sitchensis* (Sitka spruce) in primary succession may be reflected in specific belowground changes in bacterial community structure and nitrogen cycling related to the interaction of these two plants. We examined early successional alder-conditioned soils in a glacial forefield to delineate how alders alter the soil microbial community with increasing dominance. Further, we assessed the impact of late-successional spruce plants on these early successional alder-conditioned microbiomes and related nitrogen cycling through a leachate addition microcosm experiment. We show how increasingly abundant alder select for particular bacterial taxa. Additionally, we found that spruce leachate significantly alters the composition of these microbial communities in large part by driving declines in taxa that are enriched by alder, including bacterial symbionts. We found these effects to be spruce specific, beyond a general leachate effect. Our work also demonstrates a unique influence of spruce on ammonium availability. Such insights bolster theory relating the importance of plant–microbe interactions with late-successional plants and interspecific plant interactions more generally.

## Introduction

Building on long-standing perspectives that have examined ecosystem succession in terms of plant communities (Clements, 1916; Vitousek et al., 1993; Chapin et al., 1994), research is increasingly demonstrating the importance of soil microbial community succession in mediating both physical and chemical changes in ecosystem development (Nemergut et al., 2007; Schmidt et al., 2008; Knelman et al., 2014; Castle et al., 2017). In combining both perspectives, the importance of plant–microbe interactions as a driver of community assembly and ecosystem succession has come to light (Bartelt-Ryser et al., 2005; Kardol et al., 2006; Cline and Zak, 2015; Knelman et al., 2015; Yuan et al., 2016, 2017; Bueno de Mesquita et al., 2017).

Plants may exert species-specific effects on microbial communities through litter inputs, rhizodeposition, and the unique chemical and physical attributes of the root compartment (Bonito et al., 2014; Lebeis et al., 2015; Reinhold-Hurek et al., 2015; Lareen et al., 2016). Such microbial communities may then feedback on plant communities through direct effects of plant-microbe symbioses and indirect effects via changes in microbial-mediated biogeochemistry (Van Der Heijden et al., 2008; Bever et al., 2010; Ke et al., 2015; Agler et al., 2016; Van Der Heijden and Hartmann, 2016). Plant–microbe interactions have the potential to mechanistically explain not only individual plant performance in the environment, but also interspecific plant interactions (such as competition or facilitation) and community assembly across ecosystem succession (Bever, 2003; Bever et al., 2010; Fukami and Nakajima, 2013).

Past research relating plant–microbe interactions to ecosystem succession has generally focused on how soil microbiomes are conditioned by early successional species and may differentially feedback on subsequent colonization by both conspecific and heterospecific plants of the same or different successional stages (Van der Putten et al., 1993; Kardol et al., 2006, 2007; Kulmatiski et al., 2008; van de Voorde et al., 2011; Middleton and Bever, 2012; Herzberger et al., 2015; Castle et al., 2016). Related research has established the importance of both positive and negative plant-microbe feedbacks in driving plant community assembly (Bardgett et al., 2005; Bartelt-Ryser et al., 2005; Van Der Heijden et al., 2006; Bever et al., 2012; van der Putten et al., 2013). Nonetheless, beyond understanding how heterospecific plants condition soil microbial communities with implication for community assembly across succession, there remains a need to mechanistically describe how these interactions are being played out in soil bacterial community structure and related biogeochemistry at a higher resolution.

In this study, we sought to understand how interacting plants influence soil microbial communities and related function in primary succession, an interaction that may underlie past research that has shown strong effects of plant-conditioned soils on interspecific plant interactions in succession (Clein and Schimel, 1995; Schimel et al., 1996; Kardol et al., 2007). First, we examined how the increasing dominance of alder in early primary succession may control patterns of soil bacterial community structure. Second, as leaf chemistry is known to be a plant trait that has a major influence on plant–soil microbe interactions (terHorst and Zee, 2016), we used a leaf-leachate microcosm experiment to test the effect of colonizing late-succession *Picea sitchensis* (spruce) on *Alnus sinuata* (alder)-dominated, early successional soils.

The Mendenhall Glacier outside of Juneau, AK, United States, provides a primary succession chronosequence over which plant community changes follow typical successional patterns (Chapin et al., 1994; Knelman et al., 2012): early succession N-fixing alder are replaced by late-successional spruce, a transition that has long been studied in terms of plant life history traits, biotic interactions, and stochastic processes (Walker and Chapin, 1986; Walker et al., 1986; Chapin et al., 1994). In our study, we sought to examine how this hallmark turnover from alder to spruce-dominated plant communities at the Mendenhall Glacier forefield may also be reflected in belowground microbial community structure and biogeochemistry. While past research at this site has shown that alder and spruce separately harbor unique microbial communities (Knelman et al., 2012), little is known about how soil bacterial communities of increasing alder dominance may respond to spruce influence in structure and function. Building on ecological theory that suggests a stronger role of microbial communities in supporting late-successional species (Middleton and Bever, 2012; Abbott et al., 2015; Koziol and Bever, 2015) and the importance of interspecific plant–microbe interactions through succession (Kardol et al., 2006, 2007; Middleton and Bever, 2012; Fukami and Nakajima, 2013), we hypothesize that alder influenced soil bacterial communities and nutrient cycling in early succession will be susceptible to microbiome and nutrient changes driven by spruce effects, a late successional species. More specifically, we hypothesize that (1) increasing alder dominance over the natural chronosequence will lead to directional changes in the bacterial community composition including relative abundance of known plant symbionts (Rhizobiales and Actinomycetales), and (2) interspecific plant effects, mediated by late-successional spruce leachate additions, will drive bacterial composition shifts including opposite changes in the relative abundance of major bacterial taxa and plant symbionts observed under alder effects. Likewise, we expect that in altering microbial community structure of alder-conditioned soils, spruce leachate effects will also uniquely influence microbial activity as evidenced by differences in soil enzyme activity and N pools.

## Materials and Methods

### Site Description and Environmental Sampling

**FIGURE 1:**
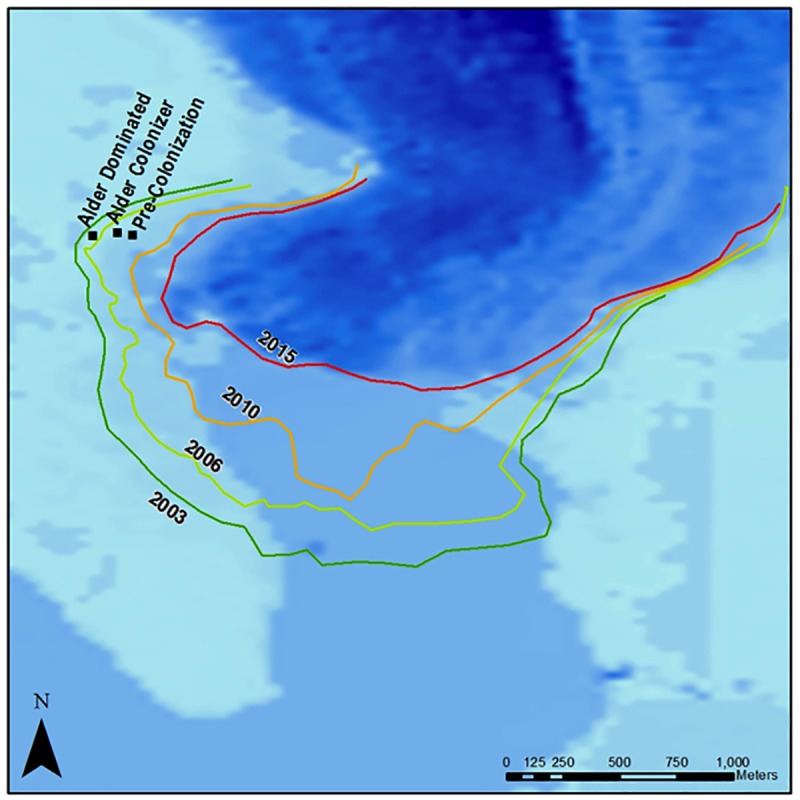
Map of Mendenhall Glacier and forefield, sampling locations, and extent of glacial cover at various years. Darkest blue color is the existing glacier, medium blue is the glacial lake, and light blue is exposed ground. Soil collection sites for increasing alder dominance are marked.

We sampled soils from the Mendenhall Glacier forefield in July 2014. The glacier forefield at the west side of the glacier terminus, where sampling took place (**Figure 1**), is the result of ongoing deglaciation since the Little Ice Age. Exposed soils are Entisols of granitic tills (Burt and Alexander, 1996). The glacier terminus is roughly 20 m above sea level with a mean annual precipitation > 2500 mm (Burt and Alexander, 1996). Soils were collected from three transects of 100 m length, at increasing distance from the glacier, representing ecologically important stages of succession: before vascular plant colonization (pre-colonization: N 58° 26.667′; W 134° 26.456′), initial alder colonized soils (alder colonizer: N 58° 26.673′; W 134° 33.397′), and alder stand soils (alder dominated: N 58° 26.665′; W 134° 33.457′) (**Figure 1**). Pre-colonization, alder-colonizer, and alder-dominated soils corresponded with approximately 7, 9, and 11 years post-deglaciation, respectively (**Figure 1**). A total of 10 samples were collected along the pre-colonization transect in unvegetated soils. A total of 10 samples were collected from the alder colonizer transect with spatially patchy alder colonizers from 45 to 60 cm tall. Here, soils were collected at the base of the alder seedling trunks, with no other vegetation within a 40 cm radius of the plant. This area was co-dominated by patchy fireweed. A total of 10 replicate soils were collected in a dense alder stand (1–1.4 m tall). Fresh clippings were collected from the alder stand plants and from young spruce in an adjacent area of > 11-year-old soils. All soils were then transported back to the laboratory in Juneau, AK, United States, and sieved to 2 mm. Subsamples for molecular analysis were placed in a freezer with dry ice and the remaining samples were placed in the fridge held at 4°C within 6 h of sampling. Vegetation was immediately dried at 60°C for 2 days. Soils were transported to Boulder on ice 2 days after collection and stored in a -70°C freezer for molecular analysis and at 4°C for soil chemistry analysis.

### Microcosm Experiment

Dried leaves from spruce and alder were ground up and 50 g of each plant was added to 800 mL of megapure water, respectively, and shaken at 350 rpm for 1 h and then allowed to sit for 14.5 h. Extracts were then filtered with burned glass microfiber GF/F 47 mm filters with 0.45 micrometer pore size. Filtered extracts were then sterile filtered with 0.22 micrometer 150 mL bottle top filters (nanopyrogenic, sterile, Corning Incorporated, Corning, NY, United States). Samples of each leachate were then run on a Shimadzu TOC-V CSN Total Organic Carbon Analyzer to evaluate non-purgeable organic carbon (NPOC) (e.g., total organic carbon) and total dissolved nitrogen (TDN). Leachates were adjusted through dilution with sterile water to create spruce and alder leachate at equivalent ending C concentrations. C:N ratios of the two leachates based on NPOC and TDN measurements were approximately equivalent. Leachate pH was tested and found to be 4.8 and 4.2 for alder and spruce leachate, respectively. For the microcosm experiment, 40 g of homogenized alder-dominated soils were added to 3 sets of 10 replicate sterilized Mason jars, for a total of 30 microcosms. In this way, all microcosms started with alder-conditioned soil of equivalent starting microbial and edaphic properties. Treatments of alder leachate, spruce leachate, and sterile H_2_O (control) were applied to the 3 sets of 10 replicate microcosms. Alder and spruce leachates were adjusted using sterilized, megapure water to ∼680 μg C/mL and ∼8 μg N/mL of leachate. A total of 5 mL of solution was added to each incubation, resulting in the addition of approximately 97 μg C/g soil and 1.1 μg N/g soil for those treated with litter leachate. The addition of alder vs. spruce leachate allowed us to not only assess the different effects of spruce and alder, but also distinguish whether effects were a general C-addition effect or specific to the heterospecific plant. Microcosms were arranged in a randomized block design for the incubation period at 18°C. Incubations were stopped after 5 days. Soils were immediately subsampled and placed in the -70°C freezer for DNA analysis as well as refrigerated at 4°C for enzyme analyses.

### Soil Edaphic Properties

For all soils from the end of the microcosm experiment, soil moisture, pH, TDN, extractable organic carbon (NPOC), and ammonium (NH4^+^) were evaluated. A subsample of each soil was dried at 100°C for 48 h to determine gravimetric soil moisture and adjust for real soil weight in all calculations. Immediately following collection of the final microcosm soil sample, 10 g of soil were extracted for 24 h in 40 mL of 2M KCl including 1 h of shaking. Extracts were filtered with Whatman no. 1 paper (Whatman Incorporated, Florham Park, NJ, United States) and frozen until analysis. NH4^+^ was measured on a BioTek Synergy 2 Multidetection Microplate Reader (BioTek, Winooski, VT, USA) and TDN/NPOC were measured on a Shimadzu TOC-V CSN Total Organic Carbon Analyzer (Shimadzu TOCvcpn, Kyoto, Japan). pH was measured with a ratio of 1.5 g dry soil: 3 mL water. Soils were shaken at 250 rpm for 1 h, allowed to equilibrate, and then tested on an Accumet Research AR10 pH meter.

### Enzyme Analysis

Enzyme activities for β-1,4-*N*-acetylglucosaminidase (NAG), and acid phosphatase (aP) were evaluated for the microcosm experiment in order to assess microbial investment in N and P acquisition under the effect of alder or spruce litter leachate. The activities of these enzymes are indicative of microbial (both bacterial and fungal) investment in the acquisition of N and P, and the limiting nature of these nutrients. Thus, changes in these decomposition enzymes both indicate shifts in soil nutrient cycling dynamics as well as microbial nutrient limitation (Sinsabaugh et al., 2008; Holden et al., 2012; Knelman et al., 2017). Enzyme activity was measured via fluorometric microplate methods (Sinsabaugh et al., 2002; Weintraub et al., 2012). The methods of Weintraub et al. (2012) were used based on a 96-well assay plate method with 1M sodium acetate buffer titrated to a pH of 7.0 and 4-methylumbelliferone standards. Approximately 1 g of refrigerated soil was used from each sample. Each sample was run with 16 analytical replicates, quench corrections, standards, and negative controls. Fluorescence was measured using a microplate reader (Thermo Labsystems, Franklin, MA, United States) at 365 nm excitation and 460 nm emission to calculate nmol activity h^-1^ g soil^-1^.

### DNA Extractions and Illumina Sequencing

MoBio’s PowerSoil DNA Isolation Kit (MO BIO Laboratories, Carlsbad, CA, United States) was used according the manufacturer’s protocol to extract genomic DNA from each sample within the environmental and microcosm study. Samples were checked for DNA purity using a NanoDrop800 and quantified using the PicoGreen method. All samples were then normalized to 2.85 ng DNA/μL. Samples were then sent to The Genomic Sequencing and Analysis Facility at University of Texas at Austin for barcoded PCR using 515F/806R primers (Hyb515F_rRNA: 5′-TCGTCGGCAGCGTCAGATGTGTATAAGAGACAGGTGYCAGCMGCCGCGGTA-3′ and Hyb806R_rRNA: 3′-TAATCTWTGGGVHCATCAGGGACAGAGAATATGTGTAGAGGCTCGGGTGCTCTG-5′), and sequencing of the 16S rRNA gene V4 regions on a Illumina MiSeq platform, according to the sequencing center protocols^1^.

### Statistical and Sequence Analysis

Using the R statistical environment (R Development Core Team, 2013) and the *pgirmess* package in R, Kruskal–Wallis ANOVA contrasts were run to assess changes in dominant bacterial taxa. First, we evaluated changes at the phyla level – as well as genera level for well known plant symbionts within orders Rhizobiales and Actinomycetales (Nemergut et al., 2011; Yeoh et al., 2017) – across the natural chronosequence with increasing alder dominance. Within those phyla that showed significant responses with increasing alder dominance, we examined relative abundances from the microcosm experiment for all genera that constituted, on average, at least 0.1% relative abundance of communities, to better understand how spruce plant-effects impacted these alder-influenced bacterial communities. We used the *ggplot2* package in R to plot these results (**Figure 3**). Using Tukey’s Honestly Significant Differences test, we examined which genera changed significantly due to alder/spruce leachate addition in comparison with control (alder-dominated) soils collected from the natural chronosequence. Enzyme activity data was checked for normality and then assessed for differences across microcosm treatments using One-Way ANOVAs. TDN, NH4^+^, and NPOC were natural log-transformed to fulfill assumptions of normality and along with pH and soil moisture were tested for differences among incubation treatments using One-Way ANOVAs and Tukey’s Honestly Significant Differences tests.

We examined bacterial community composition and diversity using the UPARSE and QIIME software packages (Caporaso et al., 2010b; Edgar, 2013). Cutadapt (Martin, 2011) was used to trim sequencing primers from the forward and reverse reads. When both the forward and reverse primers were not detected (allowed for 10% mismatches for primer search), read pairs were removed. Paired-ends were merged, demultiplexed and stored as fastq output according to the protocol of Andrei et al. (2015) in QIIME (v1.9). Operational taxonomic units (OTUs) were picked and an OTU table was constructed using UPARSE. Using the “parallel_assign_taxonomy_rdp.py” script in QIIME, taxonomy was assigned based on the Ribosomal Database Project (RDP) classifier (Wang et al., 2007) against the “13_8” Greengenes database (DeSantis et al., 2006). A phylogenetic tree was built using FastTree (Price et al., 2009) on masked pyNAST aligned sequences (Caporaso et al., 2010a). This phylogeny was then manually rooted to Archaea in Dendroscope (v3; Huson and Scornavacca, 2012). Based on OTU tables rarified to 8,490 sequences per sample, the lowest minimum sequencing depth for a sample, community dissimilarity matrices using the weighted UniFrac method (Lozupone et al., 2006, 2007) were calculated in QIIME. Permutational MANOVAs and PERMDISP, a permutational test of homogeneity of dispersion, were performed in PRIMER E on the UniFrac dissimilarity matrices (Clarke and Gorley, 2006). Mantel-like Spearman RELATE tests were performed in PRIMER E to assess the correlation between microbial community phylogenetic dissimilarity and NH4^+^. Ammonium data was ranked and converted into a dissimilarity matrix based on Euclidean distance and then compared to the UniFrac dissimilarity matrix via this test.

### Data Availability

Sequences, mapping file, and metadata have been made available via FigShare with the DOI 10.6084/m9.figshare.5278297.v1.

## Results

### Microbes Associated with Increasing Dominance of Alder across Succession

There were significant differences in microbial community structure between the three successional time points of alder soil conditioning (**Table 1**). Sequencing data showed a clear correspondence of particular microbial taxa with the increasing dominance of alder in a directional manner. Increases in the prominence of Actinobacteria, Acidobacteria, Bacteroidetes, Planctomycetes, and Alphaproteobacteria occurred with increasing soil age and alder dominance (**Table 2**). A higher resolution view of genera within a bacterial order involved with symbioses, Rhizobiales, showed that *Agrobacterium* and *Rhizobium* increase significantly in young, alder-dominated soils. Outside of increases in *Agrobacterium* and *Rhizobium*, no other genera within Rhizobiales showed significant changes with the rising influence of alder across our sampling of the natural chronosequence. Although there were increases in Actinobacteria, no statistically significant trends were observed in the relative abundance of genera within order Actinomycetales including that of the alder symbiont, *Frankia*. Betaproteobacteria show decreases with increasing prominence of alder (**Table 2**).

**Table 1:** Permutational ANOVA (PERMANOVA) analysis for natural and incubation sample contrasts.

Category	pre-colonization	Alder-colonizer	Alder-dominant
pre-colonization	–	5.749 (0.001)	7.374 (0.001)
alder-colonizer	5.749 (0.001)	–	1.890 (0.001)
alder-dominant	7.374 (0.001)	1.890 (0.001)	–
Category	control	alder leachate	spruce leachate
control	–	1.983 (0.011)	2.347 (0.001)
alder leachate	1.983 (0.011)	–	3.176 (0.001)
spruce leachate	2.347 (0.001)	3.176 (0.001)	–

*Each contrast with *t*-statistic (*P*-value)*.

**Table 2:** Mean relative abundance (SD) of major bacterial taxa and plant symbionts across succession with increasing Alder prominence.

Successional stage	Actinobacteria	Acidobacteria	Bacteroidetes	Chloroflexi	Planctomycetes	Alphaproteobacteria	Betaproteobacteria	Deltaproteobacteria	Agrobacterium	Rhizobium
Pre-colonization	8.16 (2.09)^A^	9.55 (1.21)^A^	5.56 (0.86)^A^	5.66 (2.10)^A^	2.39 (0.61)^A^	11.04 (2.67)^A^	31.76 (5.80)^A^	3.77 (0.59)	0.03 (0.02)^A^	0.0006 (0.0002)^A^
Alder-colonizer	16.74 (3.05)^B^	10.25 (1.41)^AB^	6.71 (0.99)^AB^	7.48 (1.31)^B^	6.55 (0.81)^B^	18.71 (1.92)^B^	11.81 (2.67)^B^	4.36 (0.08)	0.16 (0.07)^A^	0.009 (0.018)^B^
Alder-dominated	16.14 (1.32)^B^	11.44 (0.44)^B^	7.17 (0.23)^B^	5.39 (0.19)^A^	6.35 (0.23)^B^	18.51 (0.91)^B^	12.65 (0.40)^B^	4.33 (0.22)	0.12 (0.02)^B^	0.22 (0.03)^B^

^A,B^*Denote significant differences (*P* < 0.05)*.

### Microcosm Experiment

**FIGURE 2:**
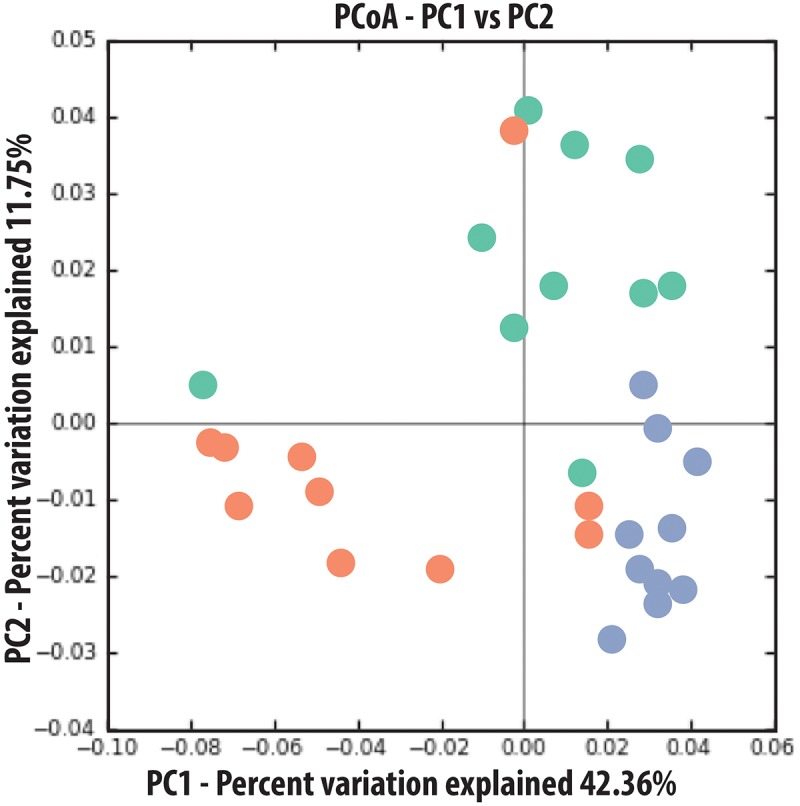
Principal Coordinates Analysis (PCoA) based on phylogenetic dissimilarity of microbial communities associated with end of experimental leachate addition microcosms. Green = control, Red = alder leachate, and Blue = Spruce leachate.

**FIGURE 3:**
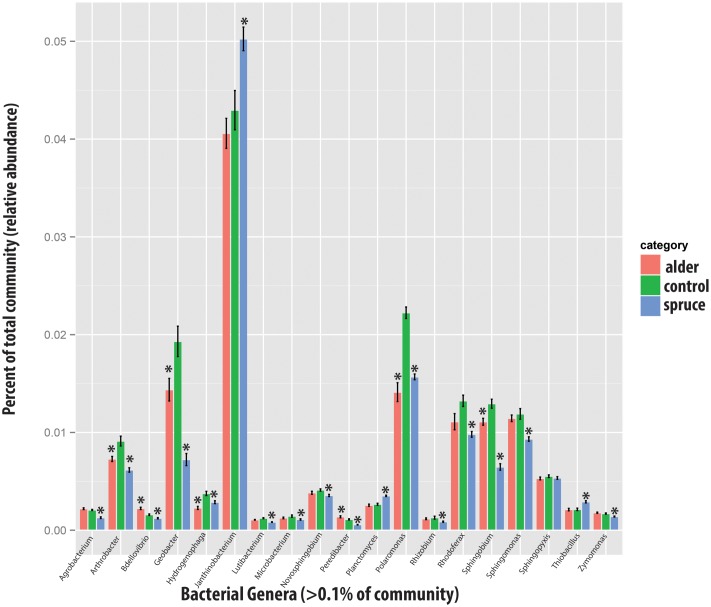
Relative change in microbial taxa at genus level with alder and spruce leachate addition in comparison with controls for major taxa [bars represent standard error; asterisk indicates significant differences (*P* < 0.05)].

Sequence analysis of the microcosm experiment showed distinct communities among treatments (**Table 1**). In particular, PERMDISP analysis showed that spruce had a greater structuring effect on microbial communities of alder-conditioned soils than other treatments, resulting in significantly less dispersion in community phylogenetic dissimilarity than both the alder leachate and the control soil communities (environmental alder-influenced soil bacterial communities) (PERMDISP, S vs. A: *P* = 0.003; S vs. control: *P* = 0.019). Controls and alder leachate soil communities did not show any statistically significant differences in the dispersion of phylogenetic community dissimilarity (PERMDISP, *P* = 0.568). Principle coordinates analysis (PCoA) based on UniFrac phylogenetic dissimilarity among communities shows grouping by treatment as well as this strong selective effect of spruce leachate on alder-conditioned microbial communities in the significant reduction of dispersion (**Figure 2**). At a higher resolution, leachate addition (both alder and spruce), in general, drove increases in the relative abundance of Acidobacteria (**Figure 3**). A differential response between alder and spruce leachate addition in relation to the control demonstrated unique spruce-driven shifts in microbial taxa as well. Out of all major taxa at the phyla level, statistically significant relative increases in Planctomycetes and Chloroflexi as well as declines in Deltaproteobacteria were associated with spruce leachate additions in comparison with microcosm controls. Further, we examined all genera within the these phyla and those that responded to increasing Alder dominance in the natural chronosequence (**Table 2**) to examine how interspecific interactions via spruce leachate addition influenced these bacteria at a higher resolution (**Figure 3**). This work showed a variety of genera in alder-influenced soils responded to spruce leachate in a significant, unique way. Some genera showed a general response to leachate addition indicating a leachate or C effect. For example, in the case of *Arthrobacter*, *Hydrogenophaga*, *Polaromonas*, and *Sphingobium*, these genera all responded with significant decreases in their relative abundance vs. control for both alder and spruce leachate addition. In other cases, Spruce uniquely drove declines in relative abundance vs. controls, where alder leachate did not (e.g., *Agrobacterium*, *Bdellovibrio*, *Lutibacterium*, *Microbacterium*, *Novosphingobium*, *Peredibacter*, *Rhizobium*, *Rhodoferax*, *Sphingomonas*, *and Zymomonas*). Finally, in the cases of *Janthinobacterium*, *Planctomyces*, and *Thiobacillus*, spruce leachate addition drove significant increases in the genera relative abundances vs. controls, whereas alder leachate addition did not.

The microcosm experiment, designed to assess the impact of spruce encroachment colonization on alder-conditioned soil communities, found no differences among treatments in soil moisture, pH, or TDN (**Table 3**). Predictably, both alder and spruce leachate treatments showed significant increases in extractable organic carbon as opposed to the control (**Table 3**), but were statistically indistinguishable from each other. Levels of NH4^+^ depended on the particular leachate addition. While both spruce and alder leachate soils showed significant decreases in NH4^+^, alder leachate soils showed significantly lower concentrations of NH4^+^ than spruce leachate soils (**Table 3**). No differences were found among treatment categories in NAG or aP enzyme activity and no correlations were found between bacterial community structure and enzyme activity. Correlations between community shifts and extractable NH4^+^ were examined, revealing a significant, albeit weak, correlation between bacterial phylogenetic community structure and soil NH4^+^ (RELATE test, Rho = 0.129, *P* = 0.026).

**Table 3:** Edaphic properties mean (SD) across ending leachate addition microcosm experiment soils.

Category	Control	Spruce leachate	Alder leachate
NPOC (mgC/kg soil)	27.88 (4.24)	33.45 (4.78)	36.48 (4.17)
TDN (mgN/kg soil)	6.48 (1.67)	6.13 (0.98)	5.74 (1.07)
NH4^+^ (mgN/kg soil)	1.89 (0.14)^A^	1.55 (0.11)^B^	1.42 (0.09)^C^
pH	7.69 (0.03)	7.72 (0.04)	7.72 (0.05)
Moisture (%)	13.62 (1.38)	13.82 (0.75)	14.08 (1.07)

^A,B,C^*Denote significant differences (*P* < 0.05)*.

## Discussion

Alder have long been described as an integral plant in driving primary succession, particularly because they have been shown to enhance soil N content and therein influence colonization by later successional species (Chapin et al., 1994; Clein and Schimel, 1995; Schimel et al., 1996). While the importance of alders in relation to plant community turnover and succession has been examined in the past (Chapin et al., 1994; Clein and Schimel, 1995; Schimel et al., 1996), research has not taken a high resolution view of how bacterial community structure of alder influenced soil changes over succession, or how interspecific interactions between these early alder soils and late successional spruce may occur via differential selection on bacterial community structure. Nonetheless, research is increasingly showing the role of plant-microbe dynamics in interspecific plant interactions and community turnover across succession (Kardol et al., 2006, 2007; Fukami and Nakajima, 2013; Abbott et al., 2015). We demonstrate in this work that over time alders directionally shape bacterial communities and that these communities are strongly altered through interspecific interactions with late successional spruce plants, as presented in our leachate addition microcosms.

### Alder Influence on Microbial Community Structure in Early Primary Succession

Here, we show a directional selection of alder on microbial community structure with colonization and increases in alder dominance across early succession. PERMANOVA analysis reveals that overall phylogenetic composition of pre-colonization, alder-colonizer, and alder-dominant soil bacterial communities are significantly different, while a higher resolution view of particular taxa associated with alder soils demonstrates that changes are consistent with alder driven effects as observed in the past at this site (Knelman et al., 2012). For example, increases in Actinobacteria attributed to alder colonization at this site are again demonstrated (**Table 2**). Alder-conditioned soils, with increases in the relative abundance of Actinobacteria, are not surprising given the symbiosis between N-fixing *Frankia* and alder (confirmed at this site) and the fact that alder may support other Actinobacteria as well (Ghodhbane-Gtari et al., 2009). Other strong changes are noted with increases in Alphaproteobacteria, including N-fixing Rhizobiales, which have been shown in past work to correspond with vegetation (including non-legumes) and plant-influenced carbon environments (King et al., 2012; Knelman et al., 2012; Cederlund et al., 2014; Yeoh et al., 2017). Conversely, Betaproteobacteria, which have been found to dominate early in succession before plant arrival (Nemergut et al., 2007; Sattin et al., 2009), strongly decrease with alder colonization in this study. In this way, our work shows how the well-documented early plant colonist, alder, drives directional shifts in bacterial community composition with increasing dominance.

### Interspecific Plant Interactions: Spruce Effects on Alder-Conditioned Soils

The microcosm experiment was designed to understand the potential for spruce effects on alder-conditioned soil bacterial communities via litter inputs, such as carbon and secondary compounds (Clein and Schimel, 1995; Schimel et al., 1996; Bowman et al., 2004). End communities of each treatment were significantly different from each other indicating the potential for a general leachate effect and heterospecific plant effect (PERMANOVA, *P* < 0.05). It is important to note that while carbon increased in both leachate-addition treatment soils, % moisture, along with NPOC, TDN, and pH, which can potentially influence microbial community structure, shows no statistically significant differences between the alder and spruce leachate addition soils.

The overall shifts in microbial community phylogenetic structure under alder and spruce leachate treatments were reflected in significant shifts in relative abundance of major taxa from the controls. Increases in the relative abundance of Acidobacteria under both spruce and alder leachate additions, for example, are indicative of a leachate effect rather than interspecific interaction. Beyond this, spruce leachate addition uniquely drove differences from controls and alder treatments with relative increases in Planctomycetes and Chloroflexi and relative decreases in Deltaproteobacteria. In this way, the spruce-specific effects – beyond simply a leachate effect on alder-conditioned soil bacterial communities – demonstrate how late successional spruce may alter bacterial communities during transitions between early and late successional plant species. Further, we point out that the starting soils were from alder-dominated soil samples, so the fact that alder leachate results in no significant effect in the cases of Planctomycetes, Chloroflexi, and Deltaproteobacteria (*Rhizobium* and *Agrobacterium* as well), reinforces that the shifts in the reported microbial taxa are spruce-specific, rather than relating to carbon addition in general.

Spruce influence was observed across a variety of bacterial genera (**Figure 3**). In some cases, these responses occurred in known plant symbionts such as *Agrobacterium* and *Rhizobium*, which show accumulation with increasing alder dominance in the natural chronosequence (**Table 2**) but decrease with Spruce influence (**Figure 3**). Such changes are of particular interest given that a directional switch in the selection of symbionts can greatly implicate plant-microbe feedbacks and the turnover of early to late successional plants (Van der Putten et al., 1993; Bever, 1994; Van Der Heijden et al., 2006; Bever et al., 2013). The spruce leachate effect specifically drove declines in these genera of Rhizobiales in contrast to the alder-conditioned control soils in the experiment and the observed pattern of increasing abundance in Rhizobiales with increasing alder-influence in the glacier forefield. Although neither plant is leguminous, past work has shown the co-occurrence of Rhizobiales with non-leguminous colonizing plants in stressful environments (King et al., 2012; Knelman et al., 2012) and Rhizobiales may be involved in asymbiotic N-fix (Buckley et al., 2007). Both Agrobacteria and Rhizobium are genera that are found in root-associated bacterial communities at global scales (Yeoh et al., 2017) and our work shows that interspecific effects lead to directional changes in the relative abundance of such symbiotic organisms as well as broader bacterial taxa and community structure. Other genera known to associate with plants endophytically, such as Janthinobacterium (Farrar et al., 2014) and Sphingomonas (Khan et al., 2014), respond to spruce leachate with significant increases and decreases respectfully as compared to alder leachate and alder-conditioned control soils. This work shows that ecologically important plant symbionts may be altered through interspecific plant interactions, though more work is needed to understand the impact on plant-microbe feedbacks.

Interestingly, we find that spruce leachate addition communities show strong and significant decreases in dispersion of bacterial community structure among communities as opposed to both the control and the alder leachate communities, which both reflect soil bacterial communities under alder influence. While we note that we would fully expect to see strong shifts in microbial community composition in response to influence by a different plant species, the change in dispersion may indicate an overall stronger structuring effect of spruce as well. This finding is consistent with research that suggests a stronger relationship between later-successional species and microbial communities and the ability of late successional species to condition early successional soils (Kardol et al., 2006; Abbott et al., 2015; Koziol and Bever, 2015).

### Spruce Leachate Effects on N Pools of Alder-Influenced Soils

While this work elucidates how interspecific interactions between early and late successional plant species may be reflected in belowground bacterial community structure, we also sought to examine functional changes in biogeochemistry associated with these community shifts. Examination of microbial enzyme activity relating to N and P cycling showed no significant change among the microcosm treatments and no connection with bacterial community composition. Levels of NH4^+^, however, depended on the particular leachate addition. Alder-leachate soils showed significantly lower concentrations of NH4^+^ than spruce leachate soils, which were both significantly lower than controls. While it is difficult to suggest a mechanism underlying these patterns due to the potential effects of N immobilization, mineralization, and nitrification (Clein and Schimel, 1995; Schimel et al., 1996), it is interesting that beyond a general leachate effect, species-specific effects drive significant differences in the availability of NH4^+^. This important shift in the cycling of inorganic N may impact transitions from early to late successional plants as has been noted in past work examining transitions of alder to poplar/spruce in ecological succession (Chapin et al., 1994; Clein and Schimel, 1995; Schimel et al., 1996). Such shifts in nutrient cycling can strongly impact plant dynamics and thus offers a mechanism by which alterations of microbial communities by early vs. late successional plants may drive successional patterns. In this study, the changes in patterns of bacterial community structure due to spruce leachate correlate with N availability. The mantel-like RELATE tests reveal a significant correlation between NH4^+^ and bacterial community structure across microcosm treatments. It is important to note that this correlation though significant is weak, but may be obfuscated by the fact that only a subset of the microbial community may be responding spruce leachate or involved with N transformations that could impact ammonium pools, for example. While further research is needed to understand how interspecific plant interactions may alter specifics of microbial community structure with implications for nutrient cycling, this research suggests it may be an important consideration in understanding the role of plant–microbe interactions as drivers of ecosystem succession.

## Conclusion

In total, this work provides important evidence that interspecific interactions between early and late successional plant species may unfold in phylogenetic compositional responses of the soil microbiome and related nutrient cycling. While laboratory experiments, such as the microcosm experiment herein, produce artificial settings for environmental microbial communities, this work is novel in showing that that early successional plant-conditioned bacterial communities are susceptible to effects of a later-successional plant species in terms of the microbiome composition. Our work shows implications for both major taxa and symbiont relative abundances within the bacterial community.

Finally, our work supports an emerging notion that plant feedbacks on the entire microbial community may have important effects on the integrated function of these communities, such as N-cycling, which can impact aboveground dynamics and ultimately transitions from early to late-successional plant species across succession. This work points to important mechanisms by which interspecific successional plants may interact belowground: interactions may unfold via changes in the soil bacterial community structure and related function (e.g., biogeochemistry) (Van Der Heijden and Hartmann, 2016). Future work may better seek to tie these mechanisms to plant performance and competition/facilitation through succession.

## Author Contributions

JK and EG designed the study; JK, EH, and SS established the field site; JK, EG, and EH completed the fieldwork; JK completed the laboratory work; JK and JP conceived of the analyses and communication; JK, MR, and PK completed the analyses; JK wrote the manuscript with insights from EG, SS, and JP and input from all co-authors.

## Conflict of Interest Statement

The authors declare that the research was conducted in the absence of any commercial or financial relationships that could be construed as a potential conflict of interest.

## Footnotes

1 https://wikis.utexas.edu/display/GSAF/Home+Page
